# Lignosulfonate and elevated pH can enhance enzymatic saccharification of lignocelluloses

**DOI:** 10.1186/1754-6834-6-9

**Published:** 2013-01-28

**Authors:** ZJ Wang, TQ Lan, JY Zhu

**Affiliations:** 1 Key Lab of Paper Science & Technology, Shandong Polytechnic University, Jinan, China; 2 US Forest Service, Forest Products Laboratory, Madison, WI, USA; 3 College of Light Industry and Food Sciences, South China University of Technology, Guangzhou, China

**Keywords:** Enzymatic hydrolysis/saccharification, Lignin/lignosulfonate, Nonspecific/nonproductive binding/adsorption, Cellulase enzymes, Pretreatment

## Abstract

**Background:**

Nonspecific (nonproductive) binding (adsorption) of cellulase by lignin has been identified as a key barrier to reduce cellulase loading for economical sugar and biofuel production from lignocellulosic biomass. Sulfite Pretreatment to Overcome Recalcitrance of Lignocelluloses (SPORL) is a relatively new process, but demonstrated robust performance for sugar and biofuel production from woody biomass especially softwoods in terms of yields and energy efficiencies. This study demonstrated the role of lignin sulfonation in enhancing enzymatic saccharification of lignocelluloses – lignosulfonate from SPORL can improve enzymatic hydrolysis of lignocelluloses, contrary to the conventional belief that lignin inhibits enzymatic hydrolysis due to nonspecific binding of cellulase.

**Results:**

The study found that lignosulfonate from SPORL pretreatment and from a commercial source inhibits enzymatic hydrolysis of pure cellulosic substrates at low concentrations due to nonspecific binding of cellulase. Surprisingly, the reduction in enzymatic saccharification efficiency of a lignocellulosic substrate was fully recovered as the concentrations of these two lignosulfonates increased. We hypothesize that lignosulfonate serves as a surfactant to enhance enzymatic hydrolysis at higher concentrations and that this enhancement offsets its inhibitive effect from nonspecific binding of cellulase, when lignosulfonate is applied to lignocellulosic solid substrates. Lignosulfonate can block nonspecific binding of cellulase by bound lignin on the solid substrates, in the same manner as a nonionic surfactant, to significantly enhance enzymatic saccharification. This enhancement is linearly proportional to the amount of lignosulfonate applied which is very important to practical applications. For a SPORL-pretreated lodgepole pine solid, 90% cellulose saccharification was achieved at cellulase loading of 13 FPU/g glucan with the application of its corresponding pretreatment hydrolysate coupled with increasing hydrolysis pH to above 5.5 compared with only 51% for the control run without lignosulfonate at pH 5.0. The pH-induced lignin surface modification at pH 5.5 further reduced nonspecific binding of cellulase by lignosulfonate.

**Conclusions:**

The results reported in this study suggest significant advantages for SPORL-pretreatment in terms of reducing water usage and enzyme dosage, and simplifying process integration, i.e., it should eliminate washing of SPORL solid fraction for direct simultaneous enzymatic saccharification and combined fermentation of enzymatic and pretreatment hydrolysates (SSCombF). Elevated pH 5.5 or higher, rather than the commonly believed optimal and widely practiced pH 4.8-5.0, should be used in conducting enzymatic saccharification of lignocelluloses.

## Background

The interactions between lignin and cellulase play a major role in enzymatic hydrolysis of lignocelluloses for sugar and biofuel production from biomass [1]. These interactions can be described as (1) lignin physical blockage to limit cellulose accessibility to cellulase [2], and (2) nonspecific adsorption or binding of cellulase enzymes to lignin [3-7]. Reported studies indicate that these two mechanisms produced negative effects on enzymatic saccharification of lignocelluloses. Pretreatment of lignocelluloses such as Organosolv [8,9] and SPORL [10] is able to partially remove lignin physical blockage by solubilizing a fraction of lignin into the hemicellulosic sugar stream (pretreatment spent liquor). However, further processing lignocelluloses to remove lignin blockage such as by delignification is not only expensive but also may not be necessary in terms of improving cellulose accessibility. It is probably more effective to address the issue of nonspecific binding of cellulase to lignin to further enhance enzymatic saccharification. One passive approach was to wash the solid fraction of pretreated lignocelluloses to eliminate nonspecific binding of cellulase to free lignin (referring to lignin separated from solid lignocellulosic substrate), as commonly described in the literature [11]. Another passive approach is to use surfactant, protein and metal compound to block bound lignin (referring to lignin retained in solid lignocellulosic substrate) and free lignin, reducing their nonspecific binding to cellulase [3,12-19]. However, washing is a significant environmental concern because the amount of water required is on the order of 10 m^3^ water/ton lignocellulose, based on pulp mill experience [19]. The applications of surfactant and protein are both expensive at the required levels.

Hydrophobic interaction has been identified as the primary driving force for protein adsorption [20]. Increasing the hydrophobicity of a substrate results in enhanced adsorption of protein or cellulase [1,21]. This suggests that nonspecific binding (adsorption) of cellulase (made of protein) to lignin will be different for lignins of different hydrophobicities. Sulfonated lignin, has good hydrophilic properties. We can hypothesize that sulfonated lignin such as lignosulfonate in the sulfite pretreatment hydrolysates (spent liquor) [9,10] may produce less nonspecific binding (adsorption) to cellulase enzymes. This hypothesis is indirectly corroborated by the excellent enzymatic digestibility of lignocellulosic substrates after sulfite pretreatment such as SPORL[10], sulfite pulping [22], and lignin sulfonation [23].

Lignosulfonate functions as a surfactant due to its strong hydrophilicity. This property has been used to develop commercial surface modification products such as dispersants and plasticizers. Therefore, we can further hypothesize that the application of lignosulfonate can reduce nonspecific binding of cellulase to the bound lignin fraction in lignocelluloses in the same manner as other surfactants to result in a gain in enzymatic hydrolysis efficiency. Furthermore, it is possible that the application of certain lignosulfonate, e.g., spent liquors from SPORL pretreatments, with low nonspecific bindings of cellulase – hypothesized in the previous paragraph – may result in a net enhancement of enzymatic saccharification of lignocelluloses. The validation or confirmation of this argument has significant scientific and practical implications for sulfite pretreatment technologies, such as SPORL [10] that has been demonstrated robust performance for sugar and biofuel production with very high yields from woody biomass including very recalcitrant softwood species [24-26]. Specifically, the separation of the SPORL pretreatment hydrolysate (spent liquor) from the SPORL pretreated solid ligno cellulosic fraction and washing of the SPORL solid fraction would not be required in order to enhance enzymatic saccharification. This can significantly simplify biorefinery process integration and save a significantly amount of water.

Previously, we demonstrated that the application of divalent metal compound, such as Ca(II) applied for neutralizing pretreatment hydrolysate, can eliminate nonspecific cellulase binding to lignosulfonate in the unwashed SPORL pretreated aspen (hardwood) solids [18,19]. The aspen lignosuflonate in the SPORL spent liquor had a low degree of lignin sulfonation because of a low sulfite loading of 3% on oven dry (od) wood used in pretreatment. This study is a step further in demonstrating the role of lignin sulfonation in enhancing enzymatic saccharification. SPORL spent liquor that contain lignosulfonate with high degree of sulfonation, produced from lodgepole pine (softwood) by SPORL at 8% sulfite loading on od wood, was directly mixed with SPORL pretreated lodgepole pine and dilute acid pretreated aspen. The objectives of the present study are: (1) to verify the two hypotheses proposed above by directly comparing enzymatic cellulose saccharification efficiencies of several SPORL-pretreated lignocellulosic substrates with and without the applications of a SPORL-pretreatment hydrolysate from lodgepole pine at different loadings; (2) to verify our previous finding [27,28]: significant improvement in cellulose saccharification when enzymatic hydrolysis of lignocelluloses is conducted at an elevated pH 5.5 – 6.2 as oppose to pH 4.8 – 5.0 as exclusively used in the literature. The elevated pH study was conducted with the application of SPORL pretreatment hydrolysate for further enhancement of saccharification efficiency. This work lays the foundation for direct simultaneous enzymatic saccharification and combined fermentation (SSFCombF) of the whole lignocellulosic slurry at high solids loadings without a separation and washing step after pretreatment [28].

## Results and discussions

### Enzymatic hydrolysis of Whatman paper with the addition of lignosulfonates

**Figure 1:**
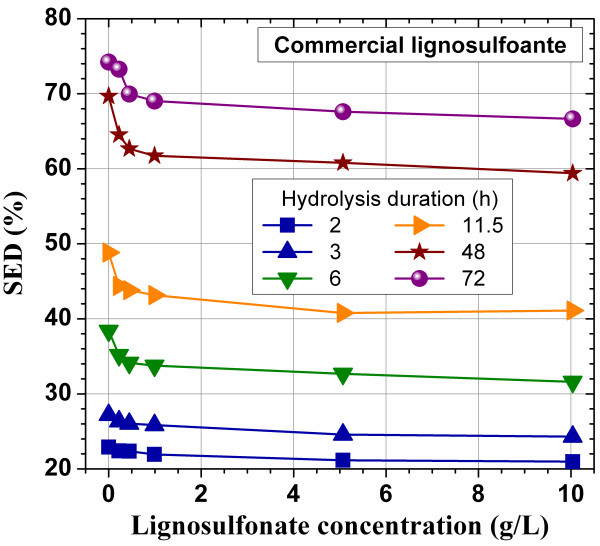
Effect of the application of a commercial lignosulfonate on substrate enzymatic digestibility (SED) of Whatman paper (pure cellulose) at cellulase loading of 15 FPU/g glucan.

Enzymatic hydrolysis of a pure cellulose substrate of Whatman paper can be inhibited by a commercial lignosulfonate as revealed in our previous study [18]. This inhibitive phenomenon was verified in this study at high concentrations upto 10 g/L using the same commercial lignosulfonate (Figure 1). The pH of the buffer solution was 4.8 and the measured pH in the hydrolysis suspension was 4.53. The reduction in substrate enzymatic digestibility (SED), defined as the percentage of glucan in the cellulosic solid substrate converted to glucose enzymatically, was rapid at low lignosulfonate concentration of less than 0.5 g/L (observable after 6 h hydrolysis) and then slowed significantly, in agreement with our previous study [18]. This reduction in enzymatic hydrolysis is due to the nonspecific lignosulfonate binding of cellulase.

**Figure 2:**
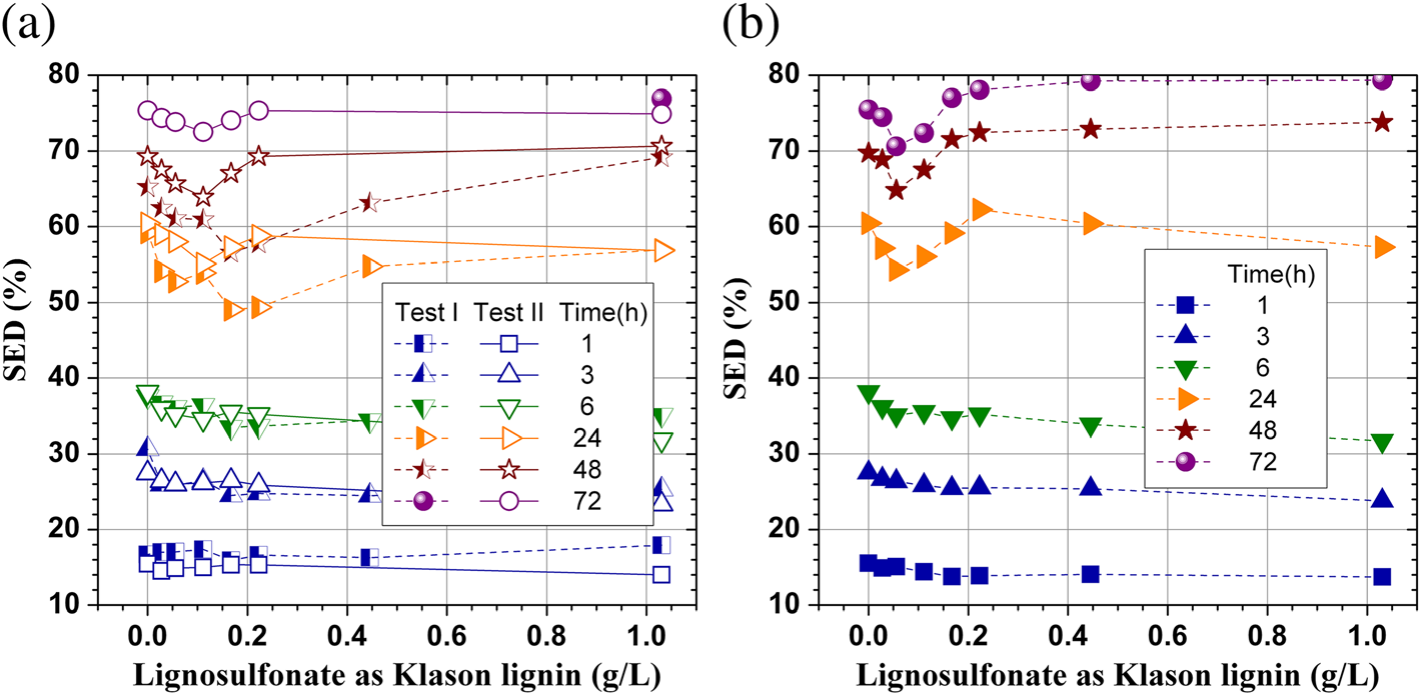
**Effect of the application of a SPORL-pretreated lodgepole pine hydrolysate (L-BD4-T85-3) on substrate enzymatic digestibility (SED) of Whatman paper (pure cellulose) at cellulase loading of 15 FPU/g glucan. (a)** L-BD4-T85-3 neutralized using NaOH to pH 4.8; **(b)** L-BD4-T85-3 neutralized using Ca(OH)_2_ to pH 4.8.

A very interesting phenomena was observed when a SPORL-pretreatment lodgepople hydrolysate (L-BD4-T85-3) was added into the enzymatic hydrolysis suspension (buffered at pH = 4.8) of Whatman paper. The SPORL hydrolysate, L-BD4-T85-3, was first neutralized from pH approximately 1.5 to 4.8 using NaOH before adding to the Whatman paper suspension. The reduction in SED was also apparent (observable after 24 h hydrolysis) and substantial at low lignosulfonate concentration of less than 0.2 g/L as Klason lignin (Figure 2a). However, SED started to recover when lignosulfoante concentration was further increased (the glucose in the pretreated hydrolysate was subtracted from the measured glucose concentration in the mixed hydrolysate in calculating SED). The recovery was also rapid with 100% recovery achieved at lignosulfonate concentration approximately 0.3 g/L as Klason lignin (Figure 2a). Although a few data points have large standard deviations for the duplicate data sets of 24 and 48 h, the results showed the similar trend. These phenomena suggest that lignosulfonate can be inhibitive to enzymatic hydrolysis of cellulose due to nonspecific binding of cellulase as previously known. The recovery of SED at high lignosulfonate concentrations can be explained first by the ionic strength effect [29]. The neutralization of the pretreatment hydrolysate using NaOH solution resulted in a Na^+^ concentration in the mixed enzymatic and pretreatment hydrolysate of 23.5 mmol/L. Our previous study found that enzymatic hydrolysis of a pretreated lignocellulosic substrate can be improved by 4% at K^+^ concentration of 2 mmol/L [19]. Lignosulfonate as a surfactant may also play a role to enhance enzymatic hydrolysis of pure cellulose [3]. The extent of this positive effect of surfactant is small for pure cellulose samples, however, and varies with the amount of surfactant applied. This explains the gradual recovery trend of SED with the amount of lignosulfonate added (Figure 2a and b). The fact that recovery of SED did not take place in Figure 1 using a commercial lignosulfonate alone without the application of ions suggests that ionic strength mechanism may play an important role for the observed recovery of glucose concentration in Figure 2a.

Our previous study indicated that a divalent metal can form complex with lignosulfoante to reduce its affinity to cellulase [18]. We conducted a separate study by neutralizing the same SPORL-pretreatment hydrolysate used in the previous set of experiments (Figure 2a) using Ca(OH)_2_. The same trend of SED reduction and recovery with the addition of lignosulfonate as discussed in the previous paragraph was observed. However, slightly higher SEDs than that for the control run without lignosulfonate were obtained after 48 hours hydrolysis when lignosulfonate concentration was greater than 0.3 g/L as Klason lignin (Figure 2b). The difference in SED between runs using NaOH (Figure 2a) and Ca(OH)_2_ neutralization (Figure 2b) were within the error margin, suggesting the lodgepole pine lignosulfonate produced using a high sulfite dosage of 8% on wood may already has very low affinity to cellulase due to its high degree of sulfonation or hydrolphlicity. The formation of lignosulfonate-Ca(II) complex on lignin nonspecific binding to cellulase is not significant.

### Enzymatic hydrolysis of a SPORL-pretreated lodgepole pine with the application of SPORL-pretreatment hydrolysates

**Figure 3:**
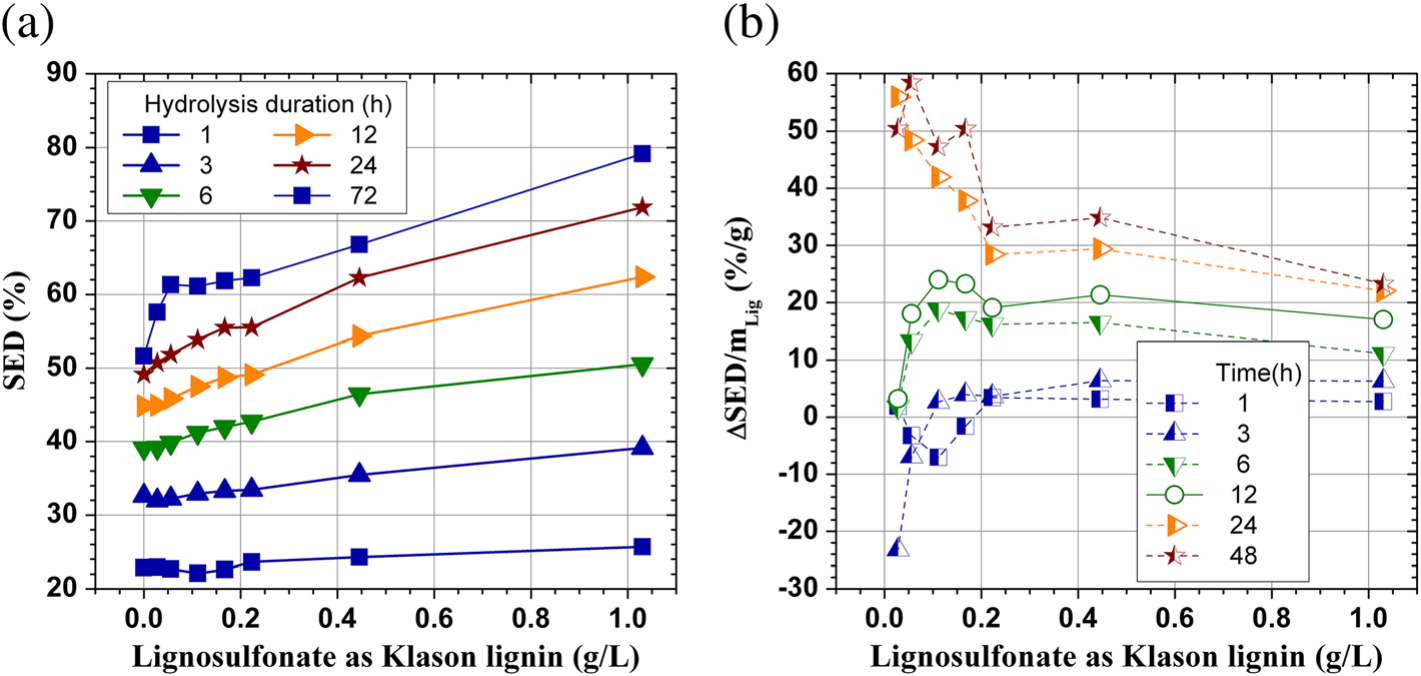
**Effect of the application of a SPORL-pretreated lodgepole pine hydrolysate (L-BD4-T85-3) on enzymatic cellulose saccharification of a SPORL-pretreated lodgepole pine (SP-BD4) at cellulase loading of 13 FPU/g glucan. (a)** Substrate enzymatic digestibility (SED); **(b)** Gains in SED per mg of lignosulfonate applied (%L/g).

The application of non-ionic surfactant can significantly reduce nonspecific binding of cellulase by lignin to enhance enzymatic hydrolysis of lignocellulsosic substrates [3,5,17]. Similarly, the application of lignosulfonate, as a surfactant, can enhance enzymatic hydrolysis of lignocellulosic substrates. A SPORL – pretreatment hydrolysate from lodgepole pine (L-BD4-T85-3) was first neutralized using NaOH to pH 4.8 and then applied to enzymatic hydrolysis of a SPORL pretreated lodgepole pine solid substrate (SP-BD4) at 2% solids (i.e., dilution with water). The results indicate cellulose conversion of SP-BD4 represented by SED increased linearly with the increase in lignosulfonate concentration (> 0.2 g/L) in the mixed hydrolysate (Figure 3a), in agreement with that reported in the literature [3,19]. Furthermore, the increase in SED, i.e., from approximately 49% to 72% at 24 h, or from 52% to 78% (by 50%) at 72 h, by applying lignosulfonate at 1.0 g/L (as Klason lignin) was also qualitatively in agreement with the gain in SED of a SO_2_ catalyzed steam exploded spruce substrate by applying non-ionic surfactant at equivalent cellulase loadings [3]. The amount of glucose in L-BD4-T85-3 (Table 1) was subtracted from the measured glucose concentration in the mixed hydrolysate for calculating SED. Lignosulfonate concentration in the mixed hydrolysate at 1.0 g/L as Klason lignin is equivalent to lignin concentration in the whole slurry of the pretreated material when hydrolyzed at 2% water insoluble solids.

**Table 1:** List of substrates produced along with the production conditions

**Sample Label**^**1**^	**Method**	**Chemical charges on wood(%)**	**T (°C)**	**Duration @ T (min)**
**Lodgepole Pine – Solidcellulosic substrates**				
SP-BD4	SPORL	H_2_SO_4_: 2.2	180	20
		NaHSO_3_: 8.0		
**Lodgepole Pine – Liquidsubstrates: Pretreatment hydrolysates (spentliquor)**				
L-BD4-T85-1	SPORL	H_2_SO_4_: 2.2	185	5
L-BD4-T85-3		NaHSO_3_: 8.0	185	25
L-BD4-T85-5			185	45
**Aspen – Solid cellulosicsubstrates**				
SP-AS	SPORL	H_2_SO_4_: 1.1	170	25
		NaHSO_3_: 3.0		
SPH-AS		H_2_SO_4_: 0	170	25
		NaHSO_3_: 3.0		
DA-AS	Dilute acid (DA)	H_2_SO_4_: 1.1	170	25

^1^SP stands for SPORL; SPH stands for SPORL with sodium bisulfite only; DA stands for dilute acid. AS stands for aspen.

The gains in SED per unit mass of lignosulfonate loading, ΔSED/m_lig_, for the results presented in Figure 3a were calculated under different lignosulfonate concentrations and various enzymatic hydrolysis durations. The results indicate negative SED gains at low lignosulfonate loadings (< 0.2 g/L as Klason lignin) and short hydrolysis duration of 6 h (Figure 3b). This again confirmed that lignosulfonate does have inhibitive effect on enzymatic hydrolysis. However, there are two competing processes, lignosulfonate adsorbed by cellulase to reduce cellulase activity and lignosulfonate serves as a surfactant to block lignin on the solid substrate to prevent cellulase adsorption on the solid substrate lignin. The second process prevails at high lignosulfonate concentrations and longer hydrolysis duration. The results also indicate that the SED gain at 48 h per unit mass of lignosulfonate, ΔSED/m_lig_, initially decreased rapidly and then decreased very slowly at lignosulfonate concentration exceeding 0.2 g/L as Klason lignin. At a lignosulfonate dosage of 1.0 g/L as Klason ligin, ΔSED/m_lig_ increased from approximately 2% L/g at hydrolysis time of 1 h to 25% L/g at 48 h. The slow-diminishing effect, i.e., ΔSED/m_lig_ decreased very slowing with the increase in lignin concentration, has significant importance for simultaneous enzymatic saccharification and combined fermentation of the enzymatic and pretreatment hydrolysates (SScombF) using the whole slurry at a high solids loadings.

**Figure 4:**
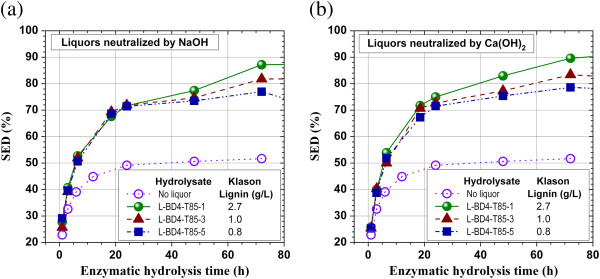
**Effect of the application of different SPORL-pretreatment lodgepole pine hydrolysates (spent liquors) produced under different pretreatment durations on SED of SP-BD4. (a)** Liquors neutralized using NaOH to 4.8; **(b)** Liquors neutralized using Ca(OH)_2_ to 4.8.

Three SPORL-pretreatment hydrolysates, i.e., L-BD4-T85-1, L-BD4-T85-2, L-BD4-T85-3, obtained from three pretreatments with different pretreatment times (Table 1) were used to further verify the positive effects of the application of lignosulfonate on enzymatic hydrolysis of lignocelluloses. Both NaOH and Ca(OH)_2_ were used to neutralize the hydrolysates to pH 4.8 before application. The results indicated that the enzymatic cellulose conversion efficiency, or SED, of a SPORL-pretreated lodgepole pine, SP-BD4, was increased by over 50% when either one of the pretreatment hydrolysate was applied (Figure 4a and b). The hydrolysate with shortest pretreatment duration of 5 min (Table 1), i.e., L-BD4-T85-1, with the highest lignosulfonate concentration of 12.6 g/L as Klason lignin (Table 1) produced highest gain in enzymatic saccharification, i.e., SED at 72 h was increased from approximately 52% to 87%, or by 67% (Figure 4a). While the hydrolysate with the longest pretreatment duration of 45 min (Table 1), L-BD4-T85-5, with lowest lignosulfonate concentration of 9.7 g/L as Klason lignin (Table 1), produced smallest gain in SED at 72 h increased by 48% (Figure 4a). This difference is mainly attributed to the differences in applied lignosulfonate dosage and in the composition of cellulase inhibitive compounds other than lignin in the pretreatment hydrolysates [30]. It is expected that a longer pretreatment duration resulted in more inhibitive compounds (i.e., furans) in the pretreatment hydrolysate, L-BD4-T85-5, and therefore a less enhancement of SED, than a shorter pretreatment. The differences in the properties of the lignosulfonates due to the varied degree of sulfonation under different pretreatment durations may also affect SED enhancement.

Hydrolysate neutralization using Ca(OH)_2_ showed consistently but only slightly increased enhancement of enzymatic saccharification at 72 h than those observed using NaOH by comparing the results in Figure 4a to those in Figure 4b. The fact that the differences in SED enhancements are pretty much within the measurement errors suggests the lignosulfonate produced at a high sulfite dosage of 8% on wood may already has a very low affinity to cellulase. The role of divalent metal to form complex with lignosulfonate to reduce lignosulfonate binding of cellulase [18,19] is not very important. This is in agreement with that observed from Figure 2a and b.

### Improved enzymatic hydrolysis of a lodgepople pine by combining with its pretreatment hydrolysate and using an elevated pH of 5.5

**Figure 5:**
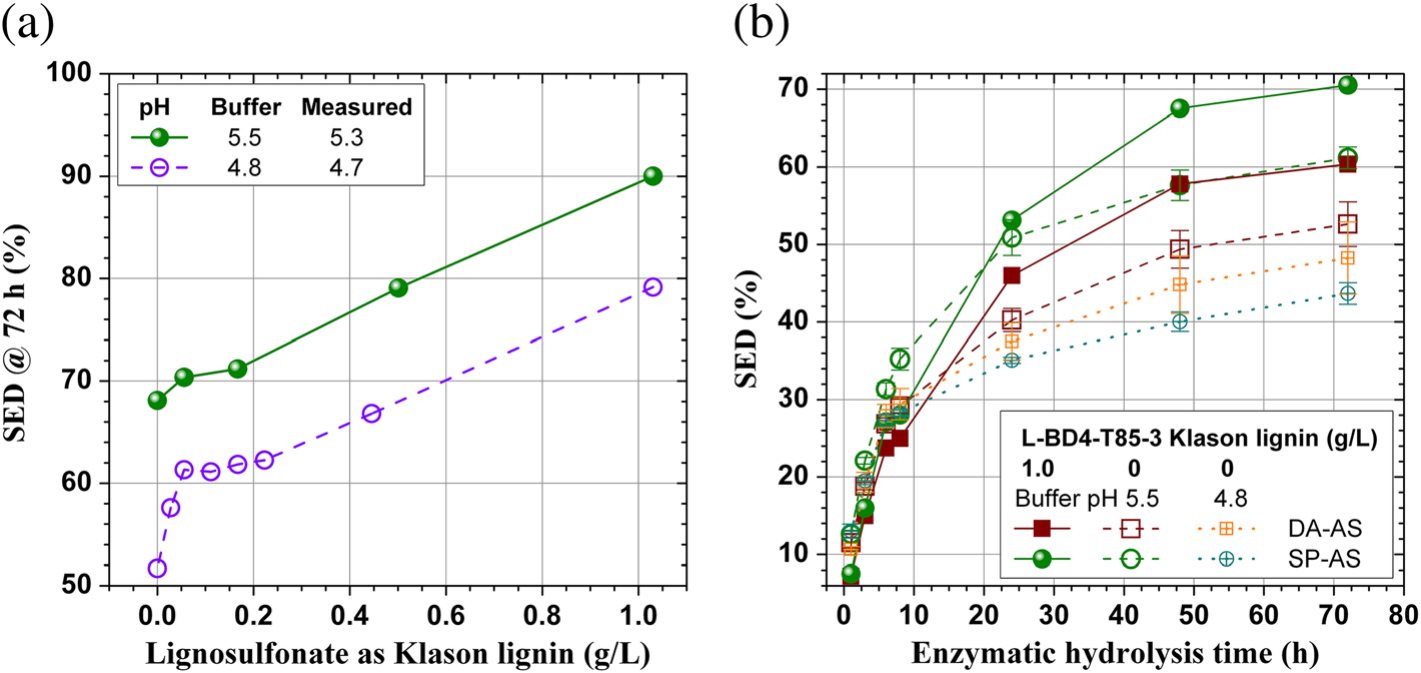
**Comparisons of SED of different pretreated solid substrates at two enzymatic saccharification pH with the applications of a SPORL-lodgepole pine hydrolysate (L-BD4-T85-3). (a)** SED of SPORL pretreated lodgepole pine (SP-BD4) at 72 h under various L-BD4-T85-3 loadings expressed as concentration of lignosulfonate in the mixed hydrolysate, Celluclase loading: 13 FPU/g glucan; **(b)** Time-dependent SEDs of SPORL- and dilute acid- pretreated aspen substrates (DA-AS, SP-AS) at a fixed L-BD4-T85-3 loading of 1.0 g/L of lignosulfonate as Klason lignin, Celluclase loading: 7.5 FPU/g glucan.

Previously we demonstrated that enzymatic saccharification efficiency of lignocelluloses can be increased significantly when hydrolysis was conducted at an elevated pH approximately 5.0 or higher for SPORL-pretreated substrates [27,28]. We mixed the same SPORL-pretreated hydrolysate (L-BD4-T85-3) with the SPORL-pretreated solid substrate (SP-BD4) to conduct enzymatic hydrolysis at an elevated buffer solution pH of 5.5. The results show that SED at 72 h increases linearly with lignosulfonate concentration in the mixed hydrolysate (Figure 5a). Furthermore, an additional gain in SED at 72 h of 10 percentage points was achieved when the pH of the buffer solution was elevated to 5.5. This demonstrates that the application of lignosulfonate at elevated pH can further enhance enzymatic hydrolysis of lignocelluloses. Cellulose saccharification efficiency or SED of 90% can be achieved at a cellulase loading of only 13 FPU/g glucan after 72 h of hydrolysis for SP-BD4. The SED of the corresponding control run (pH 4.8 without lignosulfonate) was only 51%. When a similar substrate produced from the same batch of lodgpole pine (BD4) wood chips under the identical SPORL-pertreatment conditions, a cellulase loading of 24 FPU/g glucan (15 FPU/g substrate) was required to achieve 90% cellulose conversion [31]. This suggests a savings in cellulase application of 46% can be achieved by using lignosulfonate at elevated pH. Furthermore, the increased pH is also favorable for yeast fermentations through SSFCombF.

A similar study was also conducted using a dilute acid- (DA-AS) and SPORL- (SP-AS) pretreated aspen substrates with the addition of SPORL-pretreatment hydrolysate at lignosulfonate concentration of 1.0 g/L as Klason lignin, L-BD4-T85-3, at elevated pH of 5.5. The results indicate that using a high pH alone can enhance enzymatic saccharification as represented by SED gains (Figure 5b). With the application of L-BD4-T85-3, SED decreased slightly during the first 10 hours for both substrates (by comparing the solid symbols on solid line with the corresponding open symbols on dash line), suggesting lignoslfonate can be inhibitive to enzymes. But the lignosulfonate can enhance enzymatic hydrolysis after 24 hours. The SED of both substrates after 24 hours were further enhanced at elevated pH 5.5. When compared with the control run using buffer solution pH of 4.8 without the application of pretreatment hydrolysate (L-BD4-T85-3), the SED at 72 h were increased by 25 and 62% for the DA-AS and SP-AS, respectively. The pH effect on improving enzymatic saccharification is more pronounced for the SPORL-pretreated sample compared with the dilute acid pretreated sample, and agrees with our previous study [28].

### Commercial lignosulfonate to enhance enzymatic saccharification of lignocellulose

**Figure 6:**
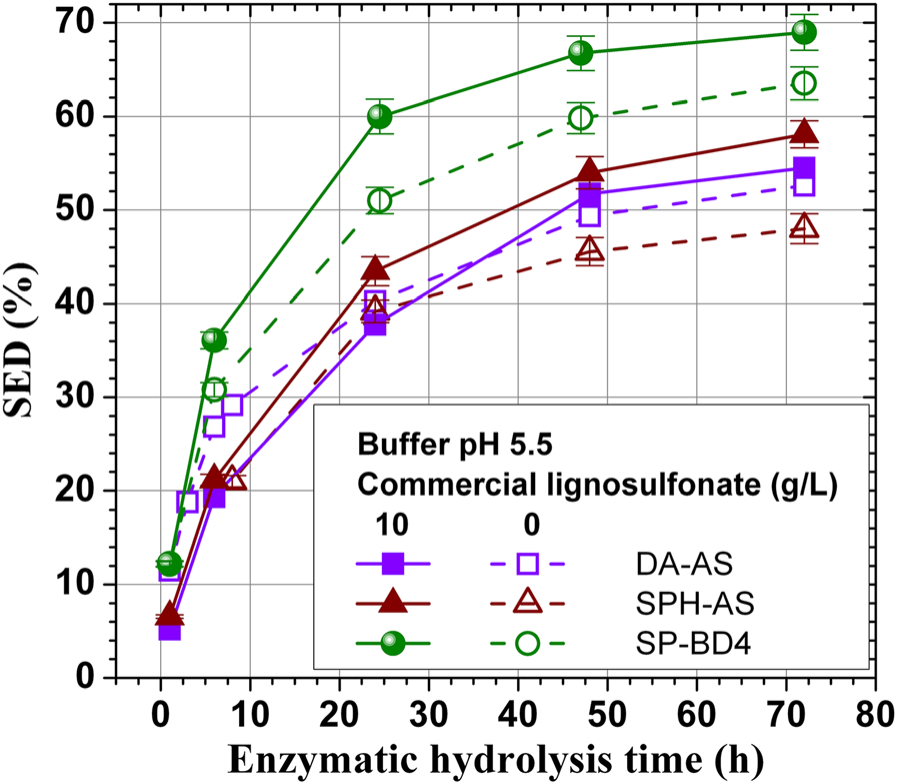
**Effect of the application of a commercial lignosulfonate on time dependent SEDs of pretreated aspen (SPH-AS, DA-AS) and a pretreated lodgepole pine (SP-BD4).** Celluclase loading 7.5 FPU/g glucan and hydrolysis buffered at pH 5.5.

The commercial lignosulfonate (D748, LignoTech USA, Rothschild, WI) was applied to enzymatic hydrolysis of dilute acid-pretreated (DA-AS) and SPORL-pretreated aspen (SPH-AS), and lodgepole pine (SP-BD4) solid substrates at lignosulfonate concentration of 10 g/L at an elevated buffer solution pH of 5.5. The SED at 72 h was increased by approximately 20 and 10% (Figure 6), respectively, for SPH-AS and SP-BD4, indicating the commercial lignosulfonate is also effective to increase enzymatic hydrolysis of lignocelluloses. However, no significant effect was observed to the dilute acid pretreated aspen (DA-AS) though slight inhibitive effect to reduce SED before 24 h and the graduate recovery of SED after 24 was observed, suggesting the interaction of lignosulfonate with the bound lignin on the lignocellulose solids also depends on the structure of the solid lignin.

## Conclusions

This study demonstrates lignosulfonate has a low affinity to cellulase. As a surfactant, certain lignosulfonate, such as lignosulfonate from SPORL pretreatment of lodgepole pine, can be applied to significantly enhance enzymatic hydrolysis of lignocelluloses. Elevated pH can also enhance enzymatic saccharification of lignocelluloses. By directly mixing SPORL pretreatment hydrolysate of a lodgepole pine with the corresponding SPORL pretreated lodgepole pine solid substrate, 90% of enzymatic saccharification of pretreated lodgepole pine can be achieved at pH 5.5 and cellulase loading of 13 FPU/g glucan, or increased by approximately 75% compared with saccharification of the washed solid substrate alone at pH 4.8. These results demonstrate the feasibility of eliminating solid substrate washing for direct simultaneous enzymatic saccharification and combined fermentation of enzymatic and pretreatment hydrolysates (SSCombF) using SPORL technology with reduced water and enzyme use.

## Materials and methods

All experiments were carried out according to the experimental process flow similar to previous described except the pretreatment spent liquor was pH adjusted before added to the solid fraction to conduct enzymatic saccharification [32].

### Materials

A lodgepole (*Pinus contorta*) tree killed by mountain pine beetle (*Dendroctonus ponderosae*) (estimated infestation age of 4 years, abbreviated BD4) was harvested from the Canyon Lakes Ranger District of the Arapaho–Roosevelt National Forest, Colorado. The details of the tree were described in our previous studies [31,33]. Fresh aspen (*Populus tremuloides*) wood logs were obtained from northern Wisconsin, USA. All wood logs were shipped to the U.S. Forest Products Laboratory, Madison, Wisconsin, and chipped using a laboratory chipper. The wood chips were then screened to remove all particles greater than 38 mm and less than 6 mm in length. The thickness of the accepted chips ranged from 1 to 5 mm. The chips were kept frozen at a temperature of about −16°C until used.

Celluclast 1.5 L and Novozyme 188 (β-glucosidase) were generously provided by Novozymes North America (Franklinton, NC). Sodium acetate buffer, sulfuric acid, and sodium bisulfite were ACS reagent grade and used as received from Sigma-Aldrich (St. Louis, MO).

### Substrate production

Several lignocellulosic solid substrates were produced from both the lodgepole pine and aspen wood chips using pretreatment methods with different process chemistries. Table 1 lists the various substrates produced along with production process conditions. Because dilute acid is a widely studied pretreatment and effective on many feedstocks except softwoods such as lodgepole pine used in this study. We also produced solid substrates from aspen (hardwood) using dilute acid and SPORL (Table 1) to evaluate the effectiveness of the application of lignosulfonate to enhance saccharfication of these aspen substrates. A laboratory wood pulping digester of capacity of 23 L was used to conduct pretreatment as described in our previous study [10]. The digester was heated by a steam jacket and rotated at 2 rpm for mixing. The oven dry (od) weight of wood chips in each pretreatment was 2 kg. The pretreatment liquid to wood ratio (L/W) was kept at 3 (v/w). The chemical charges, reaction temperature, and duration of different pretreatments are listed in Table 1.

**Figure 7:**
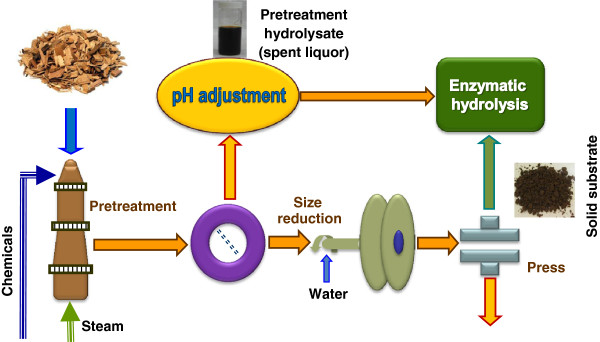
**A schematic experimental flow diagram.**

The pretreated wood chips remained intact and were separated from the pretreatment hydrolysate (hemicellulosic sugar stream) by a screen. The pretreated wood chips were disk milled using disk plates of pattern D2B-505 with a plate gap of 0.25 mm and adjusted to a refiner discharge consistency of 10% with dilution water (Figure 7). The energy consumption for disk milling was recorded as described elsewhere [34,35]. The size-reduced solids were directly dewatered to a solids content of approximately 30% by vacuum pressing in a canvas bag (as prewashing). The yields of solid substrate after washing were then determined from the weight and moisture content of the collected substrate. The chemical compositions of both the solid substrates along with the pretreatment hydrolysates were analyzed according to the methods described later in the text. The results are listed in Tables 2 and 3. It should be pointed out that the experimental flow shown in Figure 7 is to demonstrate the concept proposed in this study. The fresh water used for disk milling should be replaced by the pretreatment hydrolysate, i.e., the separation of pretreatment hydrolysate from the solids is not necessary, as practiced in our laboratory currently after the proposed concept being proven through this study.

**Table 2:** **Chemical compositions and yields of the untreated and pretreated lignocellulosic substrates listed in Table **1

**Sample label**	**K lignin (%)**	**Glucan (%)**	**Xylan (%)**	**Mannan (%)**	**Solids yield (wt%)**
Untreated wood					
BD4	28.6	41.9	5.5	11.7	100
FL	29.2	39.1	6.0	10.0	100
Aspen	20.2	45.6	16.4	1.4	100
Pretreated wood					
SP-BD4	34.7	57.4	1.5	0.6	58.6
SP-AS	28.1	66.2	1.9	0.3	58.2
SPH-AS	22.2	66.6	5.3	0.8	60.9
DA-AS	30.0	61.6	3.3	0.4	63.5

**Table 3:** **Glucose and lignosulfonate concentrations in the pretreatment hydrolysates (hemicellulosic sugar stream) listed in Table **1

**Liquid substrate**	**Glucose (g/L)**	**Lignosulfonate as Klason lignin(g/L)**
L-BD4-T85-1	2.95	12.6
L-BD4-T85-3	5.34	11.1
L-BD4-T85-5	7.18	9.7

The pH of the pretreatment hydrolysates were adjusted to 4.8 using NaOH before applied to the suspension of solid substrate to conduct enzymatic hydrolysis. For comparison purposes, the pH of the pretreatment hydrolysates were also adjusted to 4.8 using Ca(OH)_2_ in some hydrolysis experiments as indicated. The glucose was measured using the glucose analyzer described in the “Analytical methods” section.

A pure cellulosic substrate, a commercial Whatman filter paper (Grade 3, Cat No 1003 150, Whatman International, England) was also used. The manufacturer specified ash content is 0.06%. High purity lignosulfonate D748 from softwood sulfite pulping was donated by LignoTech USA (Rothschild, WI).

### Enzymatic hydrolysis

Enzymatic hydrolysis was conducted using commercial enzymes at 2% substrate solids (w/v) in 50-mL of buffer solutions on a shaker/incubator (Thermo Fisher Scientific, Model 4450, Waltham, MA) at 50°C and 200 rpm. Unless indicated, the pH of the buffer solution of sodium acetate was 4.8. Celluclast 1.5 L loadings varied between 7.5 to 13 FPU/g glucan. The ratio of Novozyme 188 (β-glucosidase) loading (in CBU) to Celluclast 1.5 L loading (FPU) was maintained at 1.5 for all experiments. Selected hydrolysis experiments were carried out in duplicates to ensure experimental repeatability. Hydrolysate was sampled periodically for glucose concentration analysis. Each data point is the average of two replicates. The average relative standard deviation was approximately 2%.

### Analytical methods

The chemical compositions of the original and pretreated biomass were analyzed by the Analytical and Microscopy Laboratory of the Forest Products Laboratory as described previously [31]. All lignocelulosic samples were Wiley milled (model #2, Arthur Thomas Co, Philedelphia, PA). The milled sample of 20 mesh (~1 mm) in size was hydrolyzed in two stages using sulfuric acid of 72% (v/v) at 30°C for 1 h and 3.6% (v/v) at 120°C for 1 h. The hydrolysate supernatant and remaining solids are then filtered through a Gooch Crucible lined with a 21 mm Whatman filter into a volumetric flask. The supernatant was used for carbohydrate analysis using high-performance anion exchange chromatography with pulsed amperometric detection (HPAEC-PAD) [36]. Klason lignin (acid insoluble) retained on the filter paper was quantified gravimetrically after drying.

The saccharides in the pretreatment hydrolysates (spent liquors) were analyzed using a Dionex HPLC system (ICS-3000) equipped with integrated amperometric detector and Carbopac™ PA1 guard and analytical columns at 20°C. Eluent was provided at a rate of 0.7 mL/min, according to the following gradient: 0 → 25 min, 100% water; 25.1 → 35 min, 30% water and 70% 0.1 M NaOH; 35.1 → 40 min, 100% water. To provide a stable baseline and detector sensitivity, 0.5 M NaOH at a rate of 0.3 mL/min was used as post-column eluent. For fast analysis, glucose in the enzymatic hydrolysate was measured in duplicate using a commercial glucose analyzer (YSI 2700S, YSI Inc., Yellow Springs, OH).

The lignosulfonate in the SPORL hydrolysate was calculated as Klason lignin based on Klason lignin yield loss through SPORL-pretreatment with the assumption that all Klason lignin losses were solublized as lignosulfonate into the SPORL hydrolysate. The amount of lignosulfonate produced from the acid soluble lignin was not accounted for.

## Competing interests

The authors declare that they have no competing interests.

## Authors’ contributions

ZJW conducted most of the experiments. TQL initiated and assisted conducting the pH effect experiments. JYZ directed the research and analyzed the data and wrote the entire paper. All authors read and approved the final manuscript.

## Authors’ information

Wang and Lan were visiting Ph. D students at the USDA Forest Service(USDA-FS), Forest Products Lab (FPL), from South China University of Technology, GuangZhou, China. Wang is currently with Key Lab of Paper Science & Technology, Shandong Polytechnic University, Jinan, China. Zhu is a Scientific Team Leader at the USDA-FS-FPL. He is a co-inventor the SPORL pretreatment process and publishes extensively in woody biomass bioconversion for biofuel, fiber, and nanocellulosic materials. Zhu is an elected Fellow of the International Academy of Wood Science (IAWS) and an officer of the Forest Products Division of the American Institute of Chemical Engineering (AIChE) and the Technical Association of the Pulp and Paper Industry (TAPPI). He serves on the editorial boards of several technical journals. This work was conducted on official government time of Zhu.
